# Validation of UBE2C protein as a prognostic marker in node-positive breast cancer

**DOI:** 10.1038/sj.bjc.6605122

**Published:** 2009-06-09

**Authors:** D Loussouarn, L Campion, F Leclair, M Campone, C Charbonnel, G Ricolleau, W Gouraud, R Bataille, P Jézéquel

**Affiliations:** 1 Service d’Anatomie Pathologique B, Hôpital G&R Laënnec, Bd J. Monod, Nantes 44805, Saint Herblain Cedex, France; 2 INSERM U892, Institut de Biologie, 9 quai Moncousu, Nantes Cedex 44035, France; 3 Unité de Biostatistique, Centre de Lutte Contre le Cancer René Gauducheau, Bd J. Monod, Nantes 44805, Saint Herblain Cedex, France; 4 Service d’Oncologie Médicale, Centre de Lutte Contre le Cancer René Gauducheau, Bd J. Monod, Nantes 44805, Saint Herblain Cedex, France; 5 Unité Mixte de Génomique du Cancer, Hôpital G&R Laënnec, Bd J. Monod, Nantes 44805, Saint Herblain Cedex, France; 6 Département de Biologie Oncologique, Centre de Lutte Contre le Cancer René Gauducheau, Bd J. Monod, Nantes 44805, Saint Herblain Cedex, France

**Keywords:** breast cancer, immunohistochemistry, Ki-67, prognostic, proliferation, UBE2C

## Abstract

**Background:**

We recently identified and validated *UBE2C* RNA as a prognostic marker in 252 node-positive (N+) breast cancers by means of a microarray study. The aim of this study was to validate UBE2C protein as a prognostic marker in N+ breast cancer by immunohistochemistry (IHC).

**Methods:**

To this end, 92 paraffin-embedded blocks were used. The impact of UBE2C IHC value on metastasis-free survival (MFS) and overall survival (OS) was evaluated and compared with Ki-67 and Nottingham prognostic index (NPI) performances.

**Results:**

In accordance with genomic data, UBE2C IHC had a significant impact both on MFS and OS (hazard ratio=6.79 – *P*=0.002; hazard ratio=7.14 – *P*=0.009, respectively). Akaike information criterion proved that the prognostic power of UBE2C IHC was stronger than that of Ki-67 (and close to that of NPI). Furthermore, multivariate analyses with NPI showed that, contrary to Ki-67 IHC, UBE2C IHC remained an independent factor, both for MFS (adjusted *P*=0.02) and OS (adjusted *P*=0.04).

**Conclusion:**

We confirmed that UBE2C protein measured by IHC could be used as a prognostic marker in N+ breast cancer. The potential predictive interest of UBE2C as a marker of proteasome activity needs further investigations.

The ubiquitin–proteasome system (UPS) is involved in many, if not all, cellular events through the regulation of protein homoeostasis and fate (Hershko *et al*, 2000). It consists of a key protein, ubiquitin; several enzymes, ubiquitin-activating enzyme (E1), ubiquitin-conjugating enzymes (E2), ubiquitin-ligating enzymes (E3), deubiquitinating enzymes and a highly sophisticated protease complex, the 26S proteasome. The latter is made up of two subcomplexes: a catalytic core particle (20S proteasome: PSMA and PSMB subunits) and one or two terminal 19S particle(s) (PSMC and PSMD subunits), which serve as proteasome activators (Tanaka, 2009). With the multitude of substrates targeted and the numerous processes involved, it is not surprising that aberrations in this pathway have been implicated in the pathogenesis of many diseases, such as cancer and, more specifically, breast cancer (Lipkowitz, 2002; Ohta and Fukuda, 2004; Mani and Gelman, 2005).

Proteomic and genomic studies conducted by our team pointed out different elements of the UPS as breast cancer prognostic markers: ubiquitin as a prognostic protein marker in node-negative (N-) patients, and *UBE2C*, *PSMA5*, *PSMB3*, *PSMB7*, and *PSMD3* as prognostic nucleic acid markers in node-positive (N+) patients, respectively (Ricolleau *et al*, 2006; Campone *et al*, 2008; Jézéquel *et al*, 2008). Our results were consistent with those of other studies (Chen and Madura, 2005; Deng *et al*, 2007). Of the 38 genes included in our 38-gene expression signature, *UBE2C* was the most highly ranked gene. Furthermore, evaluation of the prognostic power of this gene on external microarray data (more than one thousand) confirmed the robustness of this marker at the RNA level (Jézéquel *et al*, 2008). This finding was consistent with reports that underlined a strong link between *UBE2C* overexpression and the degree of tumour differentiation in many cancers (breast, lung, ovary, bladder, and glioblastomas) (Okamoto *et al*, 2003; Wagner *et al*, 2004). Furthermore, in breast cancer, an increased expression of *UBE2C* was associated with high tumour grade and cancer progression (Ma *et al*, 2003). Finally, *UBE2C* belongs to proliferative genes, which are known to constitute the majority of genes included in prognostic gene-expression signatures (Desmedt *et al*, 2008; Wirapati *et al*, 2008). The robustness of this marker may render it suitable for routine use, but the practicability of its measurement might be improved by using immunohistochemistry (IHC) staining. This technique might be preferred over nucleic acid measurement because of three advantages. First, IHC is considered as a practical approach in routine testing because of its relative inexpensiveness and straightforwardness, and is well established in standard clinical pathology laboratories. Second, IHC is applicable to paraffin-embedded tissues; there is no need for fresh, frozen, nonfixed tissue as a preferential requirement for nucleic acid quantification. Third, a more likely relevant validation consists of studying the expression level of a potential biomarker at a protein level, such as with IHC. Studies have shown that there is often discordance between levels of nucleic acids and proteins, implying that the study of both measures is important.

So the aim of this study was (1) to determine the prognostic power of UBE2C by means of IHC staining in 92 N+ breast cancer patients; (2) to compare the power of this proliferation marker with that of the reference marker in breast cancer, Ki-67, and (3) to evaluate if UBE2C added predictive accuracy to that provided by the combined prognostic index: Nottingham Prognostic Index (NPI) (Stuart-Harris *et al*, 2008).

## Materials and methods

### Patients

The study included paraffin-embedded blocks from 92 consecutive women with primary N+ breast tumours, who were diagnosed and treated primarily between April 1997 and July 2001 at the René Gauducheau Cancer Centre. Forty-three patients were also part of a previous genomic study that led to the identification of a 38-gene expression node-positive prognostic signature (Jézéquel *et al*, 2008). The median age at diagnosis was 51 years (range, 27–74 years). Informed consent was obtained from patients to use their surgical specimens and clinicopathological data for research purposes, as required by the French Committee for the Protection of Human Subjects. These patients showed no evidence of distant metastasis at the time of diagnosis. None had received chemotherapy, endocrine therapy or radiation therapy before surgery. Treatment decisions were based solely on consensus recommendations at the time of diagnosis. Patients were followed up for metastasis-free survival (MFS) (delimited by the first clinically recognised evidence of distant recurrence). All patients received FEC adjuvant chemotherapy and post-operative radiation therapy. Seventy-four received hormonotherapy (tamoxifen). Patients were followed up every 4 months during 2 years, then every 6 months during 3 years, and annually thereafter. Clinical examination, mammography and chest radiography were performed twice a year, and bone scintigraphy and liver ultrasonography annually.

### IHC

Sections (3 *μ*m) from formalin-fixed, paraffin-embedded tumours were cut and mounted on Superfrost Plus slides (VWR International, Leicestershire, UK). Following deparaffinisation in xylene, slides were rehydrated through a graded series of alcohol and placed in running water. Endogenous peroxidase activity was blocked with 3% hydrogen peroxide and methanol. Samples were steamed before incubation for antigen retrieval with 10 mM citrate buffer (pH 6.0) for UBE2C and EDTA pH 7.2 for Ki-67. Slides were incubated for UBE2C (Boston Biochem, Cambridge, UK, dilution: 1/500) and Ki-67 (Dako, Glostrup, Denmark, clone MIB1, dilution: 1/100) using a biotin-streptavidin-peroxidase detection system (Kit ChemMate, Dako). 3,3′-diaminobenzidine tetrahydrochloride (DAB) was used for the visualisation of the antibody/enzyme complex. Slides were counterstained with haematoxylin. Negative controls were included in each case by omitting the primary antibody. Ki-67 and UBE2C scores were defined as the percentage of immunostained cells divided by the total number of cells in the evaluated area. All counts were performed at a magnification of × 40 using a standard light microscope. The percentage of UBE2C and Ki-67 stained cells was evaluated individually and independently by two pathologists (DL, FL) in a double-blind manner. For each case, 250 cells were counted.

### Statistical analysis

#### Descriptive statistics

Categorical data were presented as frequencies and continuous variables were expressed as the median (range). It was also decided not to use the best, but most likely overfitted, cut-off for Ki-67 but the one calculated from 2685 patients by Viale *et al* (2008) 11% . The same one was used for UBE2C. Relationships between UBE2C IHC groups (<11% *vs* ⩾11%) and other parameters were determined by using the non-parametric Mann–Whitney test if continuous and Fisher's exact test if discrete.

#### Inter-observer reproducibility

Agreement between pathologists (DL and FL) for UBE2C and Ki-67 was verified in two ways: if continuous, by means of Spearman's correlation; if discretised (<11% *vs* ⩾11%), by means of Kappa test.

#### Correlation study between genomic and IHC data

Microarray and IHC data were available for 40 patients. Microarray characteristics and data have been deposited in the NIH Gene Expression Omnibus (Series accession number: GSE11264) according to minimum information about a microarray experiment (MIAME) (http://www.ncbi.nlm.nih.gov/geo/query/acc.cgi?acc=GSE11264) (Jézéquel *et al*, 2008). *UBE2C* RNA expression was measured by means of the two different cDNA probes named: UMGC_02270 and UMGC_06429. Correlation between *UBE2C* RNA expression and UBE2C IHC was determined by means of Spearman's test. Moreover, to be able to extrapolate results concerning UBE2C IHC impact on MFS, the previously shown prognostic value of *UBE2C* genomic value on *n*=252 patients was verified on this subsample (*n*=40) by univariate Cox regression. *UBE2C* genomic data are detailed in Supplementary Table 1 (Jézéquel *et al*, 2008).

#### Survival analysis

Categorical time from surgery to metastasis relapse (MR) (primary end point) was retained for the study. Overall survival (OS), defined as time from surgery to death from any cause, was used to reinforce MFS analysis. MFS curves were plotted according to Kaplan–Meier method and compared by means of the log-rank test. Cox's proportional hazard regression analyses were performed on UBE2C and Ki-67 expression (discretised and continuous) and on all other known prognostic parameters to assess their independent association with MFS and OS. Proportional hazard assumption was verified for the final models by means of Schoenfeld residuals study. On account of the moderate number of MR and deaths during follow-up (*n*=25/92 and 18/92, respectively), permutation tests were performed both at univariate and multivariate steps to optimise the robustness of the results.

#### Comparison and independence

Prognostic power for MFS and OS was compared for UBE2C IHC and Ki-67 IHC both discretised at retained cut-off (<11% *vs* ⩾11%) and for the NPI (classical prognostic reference) by means of Akaike information criterion (AIC) at univariate step. At multivariate step, Cox's regression analysis was used to determine whether UBE2C IHC and Ki-67 IHC (11%) added independent prognostic information to NPI.

#### Sensitivity, specificity

Receiver operating characteristic (ROC) analysis with MR and death within 7 years as a defining point was computed. The area under the curve (AUC) was used as a measure of the marker global performance in the test set. ROC curves were calculated for UBE2C IHC and Ki-67 IHC (11%) and the bio-clinical prognostic index, NPI.

All data were analysed with SAS version 9.1 (SAS Institute, Cary, NC, USA) and STATA 10 SE (StataCorp, College Station, TX, USA).

## Results

### IHC

**Figure 1:**
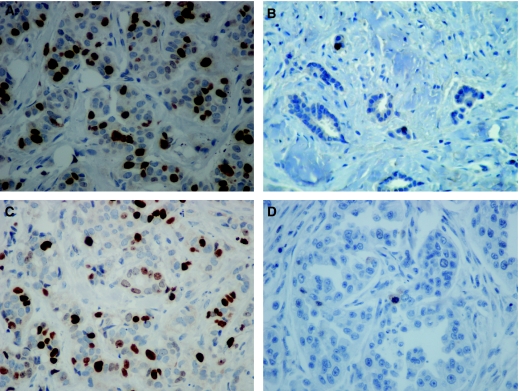
Immunohistochemistry staining of UBE2C and Ki-67 in node-positive breast carcinomas. (**A**) High expression of UBE2C with intense nuclear immunostaining of carcinoma cells. (**B**) Low expression of UBE2C with nuclear immunostaining of rare carcinoma cells. (**C**) High expression of Ki-67 with strong nuclear immunostaining of carcinoma cells. (**D**) Low expression of Ki-67 with rare immunostained carcinoma cells.

The UBE2C immunostaining was observed essentially in the nuclei of the carcinoma cells; however, in some cases, it was associated with a cytoplasmic immunostaining. The immunostaining was almost always of strong intensity (Figure 1A and B). For Ki-67 immunostaining, only nuclear staining was interpreted as positive; the intensity varied between mild and strong (Figure 1C and D). The patterns of UBE2C and Ki-67 immunostaining were similar. Exceptionally, for both Ki-67 and UBE2C, we observed nuclear immunostaining in rare normal ductal epithelial cells.

### Statistical analysis

#### Descriptive analysis

The clinical and pathological characteristics of the 92 patients are detailed in Table 1. UBE2C IHC high level (⩾11%) was significantly related to other bad prognostic parameters such as high SBR grade, number of positive nodes, negative ER, high NPI and high Ki-67 IHC level (Table 2).

#### Inter-observer reproducibility

The UBE2C and Ki-67 IHC results are robust as they showed high inter-observer reproducibility (Supplementary Table 1 and Supplementary Figure 1). As continuous parameters, Spearman's Rho showed high correlation between both pathologists (DL and FL) for both parameters (*ρ*=0.977 – *P*<0.0001 and *ρ*=0.951 – *P*<0.0001 for UBE2C and Ki-67, respectively). When discretised (<11% *vs* ⩾11%), Kappa test also showed high agreement (*κ*=0.82 – *P*<0.0001 and *κ*=0.72 – *P*<0.0001 for UBE2C and Ki-67, respectively).

#### Correlation study between genomic and IHC data

**Figure 2:**
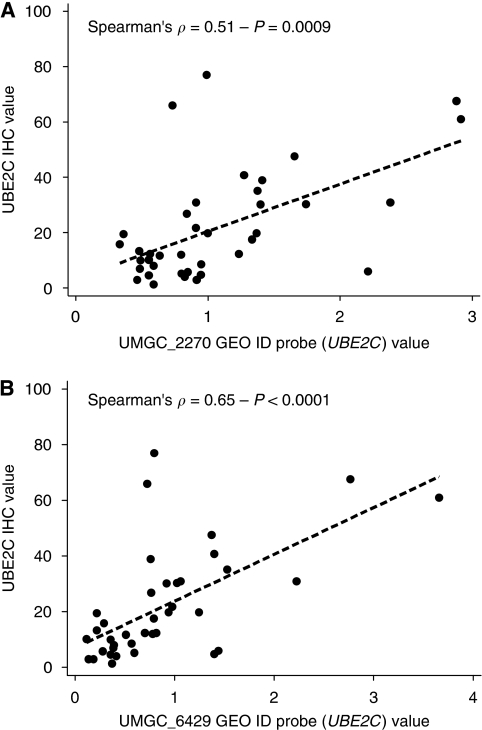
Correlation between UBE2C IHC values and UBE2C genomics value for UMGC_2270 GEO ID probe (**A**) and UMGC_6429 GEO ID probe (**B**).

Genomic and IHC data were available for 40 patients. Microarray values of the two *UBE2C* cDNA probes were significantly correlated with UBE2C IHC values (*ρ*=0.51 – *P*=0.0009 and *ρ*=0.65 – *P*<0.0001 for UMGC_2270 and UMGC_6429, respectively) (Supplementary Table 1 and Figure 2).

#### Correlation study between genomic and MFS

For the 40 patients with available UBE2C microarray data, prognostic value was confirmed for MFS prediction for both gene probes (hazard ratio (HR)=2.99, 95% confidence interval (CI))=(1.48–6.06) – *P*=0.002 and HR=2.21, 95% CI=(1.32–3.69) – *P*=0.002 for UMGC_2270 and UMGC_6429, respectively). Prognostic value was also confirmed for OS prediction for both UBE2C cDNA probes (HR=5.27, 95% CI=(2.36–11.74) – *P*<0.001 and HR=2.78, 95% CI=(1.62–4.76) – *P*<0.001 for UMGC_2270 and UMGC_6429, respectively). As UBE2C IHC and *UBE2C* genomic data were correlated, on the one hand, with each other and, on the other, with MFS, one can suggest the use of a more practicable technique (IHC) as a reliable way to measure the level of UBE2C and the transfer of this analysis to clinical routine.

### Comparison of parameters for prognostic evaluation

#### Univariate step

**Figure 3:**
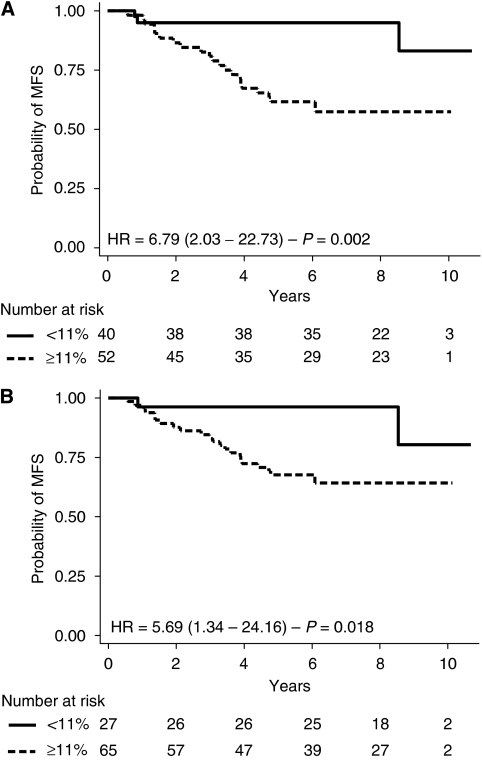
Kaplan–Meier analysis for MFS according to UBEC (**A**) and Ki-67 (**B**) IHC value.

**Figure 4:**
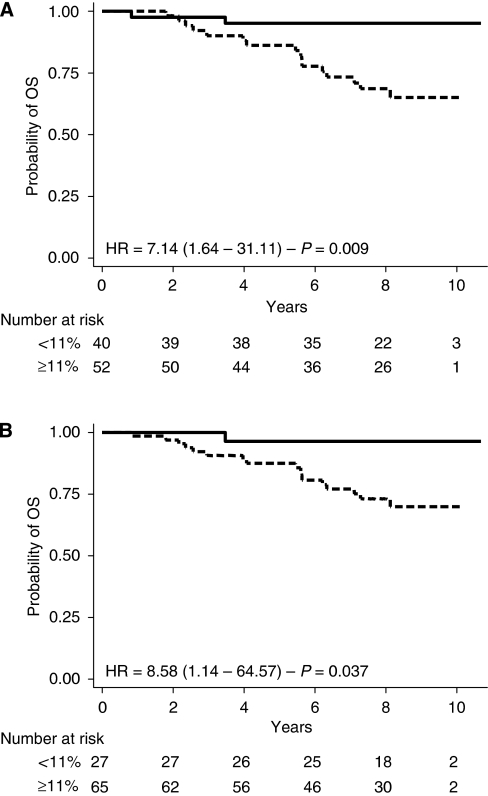
Kaplan–Meier analysis for OS according to UBEC (**A**) and Ki-67 (**B**) IHC value.

UBE2C IHC and Ki-67 IHC were both significantly related to MFS and OS (Tables 3 and 4; Figures 3 and 4). Prognostic power for MFS, measured by AIC, was greater for NPI or UBE2C IHC than for Ki-67 IHC (199.62<202.89<208.74, respectively). Prognostic power for OS, measured by AIC, was greater for UBE2C IHC or NPI than for Ki-67 IHC (146.91<147.39<149.42, respectively). Moreover, ROC analysis (MR within 7 years as a defining point) showed that AUC was 0.77, 0.74 and 0.69 for NPI, UBE2C and Ki-67, respectively (*P*=0.17). For death (7 years as a defining point), AUC was 0.74, 0.69 and 0.66 for NPI, UBE2C and Ki-67, respectively (*P*=0.43). In conclusion, univariate survival prediction's performance of UBE2C IHC is close to that achieved by NPI, and both are better than Ki-67 IHC. The results of all bio-clinical parameters are listed in Tables 3 and 4.

#### Multivariate step

Multivariate analyses showed that, for MFS and OS, discretised (11%) UBE2C IHC (but not Ki-67 IHC) remained an independent factor that added prognostic information to bio-clinical index NPI, which was the most relevant bio-clinical parameter (Tables 5 and 6, data not shown).

## Discussion

In this study, we have confirmed that increased expression of UBE2C protein was linked to poor prognosis in N+ breast cancer. Our result is in contradiction with Berlingieri *et al* (2007) who evaluated prognostic informativity of UBE2C and found no relation between this protein and the rates of overall and relapse-free survival. According to the following points, we strongly believe that UBE2C is a prognostic marker of breast cancer. First, we previously found the same result for this marker at the RNA level in a cohort of 252 node-positive breast cancer patients (Campone *et al*, 2008; Jézéquel *et al*, 2008). Second, *UBE2C* microarray data analysis of six other breast cancer genomic studies gave the same result in both N+ and N− patients (Jézéquel *et al*, 2008). Third, we found a significant correlation between *UBE2C* genomic values and UBE2C IHC values. Finally, our IHC study included more patients than did that of Berlingieri (92 instead of 74).

In regard to our recent studies and other ‘Omics’ studies, numerous components of the UPS have been found to be related to breast carcinogenesis and an unfavourable evolution in breast cancer (Chen and Madura, 2005; Ricolleau *et al*, 2006; Deng *et al*, 2007; Campone *et al*, 2008; Jézéquel *et al*, 2008). Furthermore, increased activity of the proteasome was directly linked to overexpression of UPS elements (ubiquitin enzymes and proteasomal subunits) (Chen and Madura, 2005). We can therefore hypothesise, first, that poor prognosis in N+ breast cancer is related in a large part to a high activity of the UPS, itself related to tumour high proliferative metabolism, and second, that UBE2C might be considered as a marker of proteasome activity.

On account of the central role of proteasome in protein homoeostasis and fate, whose dysregulation may lead to cancer, therapeutic strategies focused on this potential target; proteasome inhibitors could provide a new and promising class of anticancer agents (Ciechanover, 2003; Orlowsky and Dees, 2003; Sato *et al*, 2008). Since the discovery of bortezomib as proteasome inhibitor, this macromolecular protein assembly is to be considered as a therapeutic target. This drug has been approved by the Food and Drug Administration for treatment of relapsed and refractory multiple myeloma. But no effect was found in breast cancer when it was used as a single agent despite a proven efficacy when combined with several chemotherapeutic agents (Cusak, 2003). Two considerations may be advanced to explain this treatment failure. First, following the example of antioestrogens and hormone receptors, Herceptin and the HER2/neu receptor, it seems probable that the activity of the proteasome might be evaluated before any treatment with bortezomib (Marx *et al*, 2007). We propose that proteasome activity measurement could be determined by an indirect method: UBE2C IHC. To this end, we will soon compare a direct method by means of a fluorigenic substrate to UBE2C IHC (Chen and Madura, 2005). Second, according to recent study and the disappointing results of clinical trials using bortezomib as monotherapy in some solid tumours, it appears that this molecule should be used in combination. In 2006, Cardoso *et al* (2006) showed a synergy between bortezomib and trastuzumab in HER2/neu+++/++ cell lines. These results convinced the authors to conduct a phase 1 clinical trial that aimed at evaluating this drug combination.

On the basis of genomic data of a previous study, the hierarchical cluster analysis dendrogram of the 219 genes with the highest prognostic information in 252 N+ breast cancer patients showed a direct link between 2 proteasome subunit genes located in 17q12 and *ERBB2* locus, known to be a hot spot of gene amplification in breast cancer. This cluster contains: *PSMD3, PSMB3, STARD3, C17orf37 and ERBB2* (Supplementary Figure 2) (Jézéquel *et al*, 2008). Furthermore, *PSMD3* and *PSMB3* have been found to be overexpressed through gene amplification of *ERBB2* locus in numerous studies (Kauraniemi *et al*, 2003; Buness *et al*, 2007). The relation between *ERBB2* locus amplification, proteasome subunits overexpression and proteasome activity still needs to be explored, but the results exposed above may let us think that a functional link exists, and so a therapeutic approach should strongly consider this possibility.

In conclusion, in breast cancer, any new clinical trial testing bortezomib should compare bortezomib in combination (e.g., trastuzumab) *vs* the single molecule, and should include patients with proteasome high activity, which could be indirectly evaluated by UBE2C IHC staining.

## Figures and Tables

**Table 1:** Clinical and pathological characteristics of node-positive breast cancer patients

**Variable**	**All patients (*n*=92)**
Age (years)	51 (27–74)^a^
*Positive nodes*	
⩽3	66
>3	26
	
*Histology*	
IDC	72
other	20
	
*Tumour size (mm)*	25 (8–65)
ER	
+	79
−	13
	
*PR*	
+	68
−	24
	
*SBR grade*	
I	19
II	41
III	32
NPI (raw values)	5 (3.16–7.20)
	
*NPI score*	
1	7
2	51
3	34
	
*Follow-up*	
Global	8.1 (0.8–10.7)
Alive	8.1 (1.9–10.7)
Dead	4.8 (0.8–8.1)
Relapse	25
Death	18

ER=oestrogen receptor; IDC=infiltrating ductal carcinoma; NPI=Nottingham prognostic index; PR=progesterone receptor; SBR=Scarff–Bloom–Richardson.

a Median (s.d.)

**Table 2:** UBE2C IHC level and bio-clinical parameters

**Variable**	**UBE2C IHC<11% (*n*=40)**	**UBE2C IHC⩾11% (*n*=52)**	***P*-value**
Age	50.4 (8.5)^a^	54.5 (10.4)	0.030
*Histology*			
IDC−	6	12	
IDC+	34	40	0.301
			
*Histological size*			
Raw value	25 (14)	30 (14)	0.116
<20 mm	14	9	
⩾20 mm	26	43	0.088
			
*SBR*			
I	16	3	
II	18	23	
III	5	26	<0.001
			
Positive nodes			
⩽3	34	32	
>3	6	20	0.019
			
*ER*			
−	2	11	
+	38	41	0.035
			
*PR*			
−	7	17	
+	33	35	0.150
			
*Hormonotherapy*			
−	6	12	
+	34	40	0.430
			
*IHC Ki-67*			
<11%	25	2	
⩾11%	15	50	<0.001
			
*NPI*			
1	7	0	
2	27	24	
3	6	28	<0.001

ER=oestrogen receptor; IDC=infiltrating ductal carcinoma; IHC=immunohistochemistry; NPI=Nottingham prognostic index; PR=progesterone receptor; SBR=Scarff–Bloom–Richardson.

a Mean (s.d.)

**Table 3:** Metastasis-free survival univariate analyses (parametric and 1000-permutation tests)

**Variable**	**HR**	**HR 95% CI**	**Parametric *P*-value**	**1000-permutation *P*-value**
Age	1.08	1.03–1.13	0.001	<0.001
*Histology*				
IDC *vs* others	0.58	0.24–1.39	0.219	0.265
				
*Histological size*				
Raw value	1.03	1.01–1.05	0.035	0.063
⩾20 mm *vs* <20 mm	1.92	0.66–5.59	0.233	0.194
				
*SBR*				
I *vs* II *vs* III	3.15	1.59–6.23	0.001	<0.001
				
*SBR*				
I, II *vs* III	3.94	1.72–9.01	0.001	<0.001
N° positive nodes	1.28	1.17–1.40	<0.001	<0.001
				
*Positive nodes*				
1 *vs* 2 *vs* 3 *vs* > 3	2.02	1.39–2.94	<0.001	<0.001
				
*Positive nodes*				
<3 *vs* ⩾3	5.17	2.30–11.64	<0.001	<0.001
				
*ER*				
+ *vs* −	0.21	0.09–0.49	<0.001	0.001
				
*PR*				
+ *vs* −	0.42	0.19–0.94	0.035	0.043
				
*Hormonotherapy*				
+ *vs* −	0.39	0.17–0.91	0.029	0.042
				
IHC Ki-67 raw value	1.02	1.01–1.04	0.011	0.012
IHC Ki-67				
<11% *vs* ⩾11%	5.69	1.34–24.2	0.018	0.002
IHC UBE2C raw value	1.03	1.01–1.04	0.001	<0.001
IHC UBE2C				
<11% *vs* ⩾11%	6.79	2.03–22.73	0.002	<0.001
				
NPI raw value	2.81	1.84–4.28	<0.001	<0.001
NPI score				
1 *vs* 2 *vs* 3	5.45	2.33–12.71	<0.001	<0.001
NPI				
1, 2 *vs* 3	5.85	2.44–14.04	<0.001	<0.001

CI=confidence interval; ER=oestrogen receptor; HR=hazard ratio; IDC=infiltrating ductal carcinoma; IHC=immunohistochemistry; NPI=Nottingham prognostic index; PR=progesterone receptor; SBR=Scarff–Bloom–Richardson.

**Table 4:** Overall survival univariate analyses (parametric and 1000-permutation tests)

**Variable**	**HR**	**HR 95% CI**	**Parametric *P*-value**	**1000-permutation *P*-value**
Age	1.06	1.01–1.12	0.026	0.022
*Histology*				
IDC *vs* others	0.48	0.18–1.27	0.139	0.149
				
*Histological size*				
Raw value	1.02	0.99–1.05	0.133	0.157
<20 mm *vs* ⩾20 mm	2.97	0.68–12.94	0.147	0.090
				
*SBR*				
I *vs* II *vs* III	3.97	1.67–9.47	0.001	<0.001
				
*SBR*				
I, II *vs* III	5.93	2.08–16.89	0.001	<0.001
No positive nodes	1.28	1.15–1.42	<0.001	<0.001
				
*Positive nodes*				
1 *vs* 2 *vs* 3 *vs* > 3	1.81	1.19–2.74	0.005	0.002
				
*Positive nodes*				
<3 *vs* ⩾3	3.89	1.53–9.89	0.004	0.002
				
*ER*				
+ *vs* −	0.18	0.07–0.47	<0.001	<0.001
				
*PR*				
+ *vs* −	0.51	0.19–1.37	0.182	0.210
				
*Hormonotherapy*				
+ *vs* −	0.34	0.13–0.92	0.034	0.037
IHC Ki-67 raw value	1.03	1.01–1.05	0.001	0.005
				
*IHC Ki-67*				
<11% *vs* ⩾11%	8.59	1.14–64.57	0.037	0.003
IHC UBE2C raw value				
	1.03	1.01–1.05	<0.001	0.002
				
*IHC UBE2C*				
<11% *vs* ⩾11%	7.14	1.64–31.11	0.009	<0.001
				
NPI raw value	2.70	1.66–4.41	<0.001	<0.001
NPI scores				
1 *vs* 2 *vs* 3	4.48	1.74–11.52	0.002	<0.001
				
*NPI*				
1, 2 *vs* 3	4.74	1.77–12.69	0.002	<0.001

CI=confidence interval; ER=oestrogen receptor; HR=hazard ratio; IDC=infiltrating ductal carcinoma; IHC=immunohistochemistry; NPI=Nottingham prognostic index; PR=progesterone receptor; SBR=Scarff–Bloom–Richardson.

**Table 5:** Metastasis-free survival analyses for UBEC2C and Ki-67 adjusted for NPI (parametric and 1000-permutation tests)

**Variable**	**HR**	**HR 95% CI**	**Parametric *P*-value**	**1000-permutation *P*-value**
NPI^a^				
1, 2 *vs* 3	3.65	1.44–9.24	0.006	0.002
				
*UBE2C IHC*				
<11% *vs* ⩾11%	3.83	1.06–13.81	0.041	0.024
				
*NPI* b				
1, 2 *vs* 3	4.30	1.68–11.03	0.002	0.001
				
*Ki-67 IHC*				
<11% *vs* ⩾11%	2.65	0.56–12.59	0.220	0.195

CI=confidence interval; HR=hazard ratio; IHC=immunohistochemistry; NPI=Nottingham prognostic index.

a NPI adjusted for UBE2C IHC.

b NPI adjusted for Ki-67 IHC.

**Table 6:** Overall-survival analyses for UBEC2C and Ki-67 adjusted for NPI (parametric and 1000-permutation tests)

**Variable**	**HR**	**HR 95% CI**	**Parametric *P*-value**	**1000-permutation *P*-value**
*NPI* a				
1, 2 *vs* 3	2.86	1.01–8.07	0.047	0.038
				
UBE2C IHC				
<11% *vs* ⩾11%	4.52	0.96–21.32	0.057	0.040
				
NPI^b^				
1, 2 *vs* 3	3.15	1.14–8.76	0.028	0.029
				
Ki-67 IHC				
<11% *vs* ⩾11%	4.98	0.61–40.56	0.133	0.074

CI=confidence interval; HR=hazard ratio; IHC=immunohistochemistry; NPI=Nottingham prognostic index.

a NPI adjusted for UBE2C IHC.

b NPI adjusted for Ki-67 IHC.
