# Supplementary material for: Validation of UBE2C protein as a prognostic marker in node-positive breast cancer
Source: Br J Cancer. 2009 Jun 9;101(1):166–73. doi: 10.1038/sj.bjc.6605122 (PMC2713693; doi:10.1038/sj.bjc.6605122)
Supplement: Supplementary Table 1 and Supplementary Figure 1 and 2 [file 6605122x1.doc]

**Supplementary Table 1** UBE2C IHC (DL and FL), Ki-67 IHC (DL and FL) and *UBE2C* genomic values

| ID | IHC (%) | | | | *UBE2C* genomic values | |
| --- | --- | --- | --- | --- | --- | --- |
|  | UBE2C DL | UBE2C FL | KI67 DL | KI67 FL | UMGC_ 2270 | UMGC_ 6429 |
| B99006920 | 15.2 | 16.4 | 36.8 | 36 | 0.332287291 | 0.289972573 |
| B00002198 | 18.6 | 20.4 | 20 | 27.2 | 0.360512033 | 0.217267408 |
| B99006755 | 3 | 3 | 2 | 2 | 0.463798678 | 0.183916146 |
| B99008854 | 12.4 | 14.2 | 10 | 10.4 | 0.479107012 | 0.218491068 |
| B99002816 | 6.4 | 7.6 | 7.6 | 6 | 0.485421351 | 0.38212804 |
| B99003886 | 8.8 | 11.2 | 8 | 5.6 | 0.489715073 | 0.354971179 |
| B00006355 | 11.6 | 10.8 | 12.8 | 12 | 0.552186055 | 0.115892236 |
| B00000290 | 5.6 | 3.6 | 6 | 4 | 0.552704942 | 0.357054078 |
| B00001097 | 10 | 28.4 | 10 | 13.2 | 0.561408779 | 0.6991718 |
| B00001097 | 10 | 28.4 | 10 | 13.2 | 0.561408779 | 0.6991718 |
| B9710909 | 8 | 8 | 40 | 46.4 | 0.58819974 | 0.388694209 |
| B98005439 | 1.6 | 1 | 13.2 | 14 | 0.589639026 | 0.370860832 |
| B98009505 | 12.2 | 11.2 | 29.6 | 20 | 0.633753984 | 0.511813232 |
| B00000525 | 65.2 | 66.8 | 60 | 72 | 0.730615216 | 0.72560206 |
| B00006394 | 12.4 | 11.6 | 22 | 26 | 0.797924614 | 0.779646789 |
| B98011415 | 5.6 | 4.8 | 20 | 22.4 | 0.799442662 | 0.594755349 |
| B98011077 | 3.6 | 4.4 | 6 | 10 | 0.823727148 | 0.414965608 |
| B98008014 | 26 | 27.6 | 64 | 66.4 | 0.840641646 | 0.764133802 |
| B00003405 | 5.8 | 5.8 | 6 | 7.6 | 0.848082356 | 0.27712075 |
| B98004861 | 20 | 23.6 | 42 | 46.4 | 0.908773844 | 0.976584464 |
| B00007417 | 31.6 | 30.4 | 40 | 44.8 | 0.90953761 | 1.06052132 |
| B00003758 | 3.6 | 2.4 | 0.4 | 1.6 | 0.915530928 | 0.137004874 |
| B00003758 | 3.6 | 2.4 | 0.4 | 1.6 | 0.915530928 | 0.137004874 |
| B00004846 | 4.4 | 5.2 | 6.8 | 6 | 0.945654266 | 1.397505593 |
| B00005791 | 8 | 9.2 | 26.4 | 30.4 | 0.949307707 | 0.56704964 |
| B99006753 | 77.2 | 76.8 | 58 | 74 | 0.989256411 | 0.79446361 |
| B00006526 | 20 | 19.6 | 20.4 | 24.8 | 0.999482649 | 0.938944861 |
| B00006042 | 11.6 | 13.2 | 32 | 33.6 | 1.233442495 | 0.815672492 |
| B00000405 | 41.2 | 40.4 | 50.4 | 52.8 | 1.273544655 | 1.397970518 |
| B99004759 | 17.2 | 18 | 18.4 | 25.2 | 1.334318726 | 0.790332351 |
| B99005600 | 19.2 | 20.4 | 30 | 16.6 | 1.367164573 | 1.243455095 |
| B99004125 | 35.2 | 35.2 | 24 | 26 | 1.373734536 | 1.528466011 |
| B99004704 | 28.4 | 32 | 50 | 52.6 | 1.396734694 | 0.919189475 |
| B99004644 | 38 | 40 | 28.4 | 55.2 | 1.411850505 | 0.756654118 |
| B99009089 | 47 | 30 | 55.6 | 65.6 | 1.656667775 | 1.370541009 |
| B99010426 | 28 | 32.6 | 38 | 41.2 | 1.741202207 | 1.02399568 |
| B01002481 | 6.8 | 5.2 | 4 | 4 | 2.210267142 | 1.441629844 |
| B98001264 | 28 | 34 | 31.2 | 16 | 2.380484432 | 2.226967585 |
| B01000926 | 66.8 | 68.4 | 86 | 98.8 | 2.880205391 | 2.76648534 |
| B01003171 | 60 | 62 | 22 | 30.8 | 2.91568768 | 3.655941327 |
| B00000139 | 4 | 4 | 5.2 | 3.6 | nd | nd |
| B00000245 | 18.4 | 19.6 | 20 | 40 | nd | nd |
| B00000605 | 17.6 | 19.2 | 16 | 16.8 | nd | nd |
| B00000631 | 81.6 | 80 | 96 | 99.2 | nd | nd |
| B00000758 | 80 | 70 | 66.4 | 64 | nd | nd |
| B00000977 | 18 | 26 | 14 | 12 | nd | nd |
| B00001250 | 14 | 10 | 10 | 10 | nd | nd |
| B00001253 | 24 | 29.2 | 32 | 36.8 | nd | nd |
| B00001934 | 47.6 | 44.4 | 62.8 | 54 | nd | nd |
| B00001964 | 10.8 | 10.8 | 8 | 13.6 | nd | nd |
| B00001964 | 10.8 | 10.8 | 8 | 13.6 | nd | nd |
| B00002171 | 28.4 | 35.6 | 24 | 29.6 | nd | nd |
| B00003186 | 13.2 | 13.6 | 14.6 | 16.4 | nd | nd |
| B00003469 | 13.2 | 18 | 26 | 18 | nd | nd |
| B00003591 | 8.8 | 10.4 | 9 | 9.4 | nd | nd |
| B00004907 | 33 | 27.6 | 30.4 | 24 | nd | nd |
| B00004978 | 55.6 | 54 | 34.8 | 30.4 | nd | nd |
| B00004996 | 8.8 | 7.2 | 29.6 | 28 | nd | nd |
| B00005243 | 4.4 | 5.2 | 1.8 | 1 | nd | nd |
| B00005269 | 6.4 | 1.6 | 16.4 | 8.4 | nd | nd |
| B00005278 | 11.2 | 8.4 | 5 | 5.8 | nd | nd |
| B00005625 | 4 | 5.2 | 8.4 | 10.8 | nd | nd |
| B00005868 | 14.8 | 15.6 | 18.8 | 18 | nd | nd |
| B00006099 | 10.4 | 8.8 | 14 | 4 | nd | nd |
| B00006209 | 11.6 | 11.6 | 12.8 | 20.8 | nd | nd |
| B00006211 | 11.2 | 10 | 20.4 | 22.4 | nd | nd |
| B00006349 | 16.4 | 14.4 | 68 | 64.4 | nd | nd |
| B00006410 | 91.6 | 88 | 64.4 | 58 | nd | nd |
| B00006919 | 38 | 39.6 | 60 | 80 | nd | nd |
| B00006921 | 10 | 10.4 | 16 | 4 | nd | nd |
| B00006925 | 8.6 | 9.4 | 6 | 10.8 | nd | nd |
| B00007049 | 6.8 | 6 | 26.4 | 37.6 | nd | nd |
| B00007510 | 3.2 | 3.2 | 8.4 | 10.8 | nd | nd |
| B00007706 | 7.2 | 8.8 | 12 | 12.8 | nd | nd |
| B00007782 | 6 | 6.4 | 4.8 | 6 | nd | nd |
| B00007876 | 2 | 2.4 | 6 | 2 | nd | nd |
| B00008135 | 9.2 | 8.4 | 14.8 | 10 | nd | nd |
| B00008609 | 7.2 | 6 | 6 | 18 | nd | nd |
| B00010041 | 40.8 | 52 | 54.8 | 64.4 | nd | nd |
| B00010086 | 4.8 | 6.8 | 2.8 | 4.4 | nd | nd |
| B00010186 | 16.8 | 18.4 | 27.6 | 18.8 | nd | nd |
| B00010245 | 5.2 | 6.4 | 17.2 | 6.8 | nd | nd |
| B00010793 | 9.2 | 10.8 | 12.8 | 12 | nd | nd |
| B00010966 | 20.8 | 20 | 36.6 | 38.6 | nd | nd |
| B01002165 | 47.2 | 48.4 | 28 | 32.4 | nd | nd |
| B01003873 | 10.8 | 12.4 | 27.2 | 23.6 | nd | nd |
| B01005857 | 14 | 12 | 14.4 | 24 | nd | nd |
| B01006242 | 7.2 | 5.6 | 16.4 | 6 | nd | nd |
| B97004720 | 2.2 | 2.6 | 5 | 5 | nd | nd |
| B98001653 | 0.2 | 0.6 | 1.6 | 2.4 | nd | nd |
| B98008735 | 11 | 12.2 | 30 | 34.4 | nd | nd |
| B99009506 | 31.6 | 32 | 38.4 | 36 | nd | nd |

ID: patient identification; nd: not determined

**Supplementary figure 1** Inter-observer reproducibility (DL and FL) for UBE2C IHC value (**A**) and Ki-67 IHC value (**B**)

| **A** |
| --- |
| **B** |

**Supplementary figure 2** Hierarchical cluster analysis dendrogram detail of unsupervised analysis; 219 node-positive tumour samples (Jézéquel *et al*, 2009) were grouped by similarity of the expression pattern by average linkage clustering by using the software CLUSTER 3.0; similarity metric used was Spearman rank correlation and the gene expression values were centred by median. *UBE2C* and *ERBB2* clusters are displayed in rectangles.

*UBE2C* cluster

*ERBB2*  cluster
